# Exploring omics strategies for drug discovery from *Actinomycetota* isolated from the marine ecosystem

**DOI:** 10.3389/fphar.2025.1634207

**Published:** 2025-08-15

**Authors:** Manik Prabhu Narsing Rao, Syed Raziuddin Quadri, Manda Sathish, Ngoc Tung Quach, Wen-Jun Li, Arinthip Thamchaipenet

**Affiliations:** ^1^ Instituto de Ciencias Aplicadas, Facultad de Ingeniería, Universidad Autónoma de Chile, Centro de Investigación e Innovación, Huechuraba, Chile; ^2^ Department of Medical Laboratory Technology, Faculty of Applied Medical Sciences, Northern Border University, Arar, Saudi Arabia; ^3^ Centro de Investigación de Estudios Avanzados del Maule, Vicerrectoría de Investigación y Postgrado, Universidad Católica del Maule, Talca, Chile; ^4^ Institute of Biotechnology, Vietnam Academy of Science and Technology, Hanoi, Vietnam; ^5^ Department of Genetics, Faculty of Science, Kasetsart University, Bangkok, Thailand; ^6^ State Key Laboratory of Biocontrol, Guangdong Provincial Key Laboratory of Plant Resources and Southern Marine Science and Engineering Guangdong Laboratory (Zhuhai), School of Life Sciences, Sun Yat-Sen University, Guangzhou, China; ^7^ Omics Center for Agriculture, Bioresources, Food, and Health, Kasetsart University (OmiKU), Bangkok, Thailand

**Keywords:** marine ecosystem, marine *Actinomycetota*, omics approaches, drug discovery, culture-independent analysis

## Abstract

Marine *Actinomycetota* are prolific producers of diverse bioactive secondary metabolites, making them vital for drug discovery. Traditional cultivation and bioassay-guided isolation techniques often lead to the rediscovery of the same compounds, revealing the limitations of these traditional approaches and emphasizing the need for more advanced methods. The emergence of omics technologies such as genomics, metagenomics, transcriptomics, and metabolomics has dramatically enhanced the ability to investigate microorganisms by providing detailed insights into their biosynthetic gene clusters, metabolic pathways, and regulatory mechanisms. These comprehensive tools facilitate the discovery and functional analysis of new bioactive compounds by revealing the genetic blueprints underlying their biosynthesis. Omics and function-driven techniques like heterologous expression, analytical techniques (including high-resolution mass spectrometry and nuclear magnetic resonance spectroscopy), and culture condition optimization have enabled access to previously silent or cryptic gene clusters, expanding the chemical diversity available for exploration. This review emphasizes the integration of omics-based insights with function-driven methodologies and innovative culture techniques, forming a holistic approach to unlock the extensive biosynthetic capabilities of marine *Actinomycetota*. Combining these strategies holds great promise for discovering new marine-derived compounds with potential therapeutic applications.

## Introduction

The phylum ‘*Actinobacteria*’ stands out as one of the largest bacterial groups, comprising Gram-positive bacteria known for their high GC content and exhibiting a wide array of morphologies (Williams and Vickers, 1988). Its early taxonomic arrangement was established by Stackebrandt et al. (1997) through the delineation of the class *Actinobacteria*. Presently, the phylum is categorized into six classes, 46 orders, and 79 families. Notably, recent advancements have led to the inclusion of 16 new orders and 10 new families (Salam et al., 2020). Recently, the International Code of Nomenclature of Prokaryotes (ICNP) incorporated the rank of phylum, with phylum names required to be derived from the name of a genus serving as its nomenclatural type and utilizing the suffix “*-ota*” for such names (Oren et al., 2021). In this regard, the phylum name *Actinomycetota* was proposed with *Actinomyces* as the type genus (Oren and Garrity, 2021).

*Actinomycetota* gained significant attention following the discovery of streptomycin (Schatz et al., 2005). Since then, they have emerged as a crucial antibiotic reservoir, contributing to the production of nearly two-thirds of all antibiotics produced by microorganisms (Liu et al., 2016; Narsing Rao and Li, 2022). The marine environment, which makes up 70% of the biosphere, is the main habitat on earth for a wide variety of creatures, including microorganisms (Sarkar and Suthindhiran, 2022). Microbes in the marine environment evolve various adaptation mechanisms due to the intricate nature of their surroundings, leading to distinctive physiological and metabolic characteristics (Siro et al., 2023). The initial evidence supporting the presence of marine *Actinomycetota* emerged with the discovery of *Rhodococcus marinonascens*, marking the pioneering characterization of the first species within the *Actinomycetota* group in a marine ecosystem (Helmke and Weyland, 1984). *Actinomycetota* are abundant in the marine environment by virtue of their remarkable ability to acclimate to extreme conditions and play a crucial role in the synthesis of a broad variety of compounds (Sarkar and Suthindhiran, 2022). Natural products derived from marine *Actinomycetota* exhibit distinctive structural characteristics that were rarely or never encountered in the strains isolated from terrestrial sources (Bister et al., 2004; Zotchev, 2012). Drug development has typically relied on the “function to gene” technique, which entails extracting, cloning, expressing, and characterizing a gene of interest (Debouck and Metcalf, 2000). Despite being challenging and time-consuming, this method resulted in well-defined therapeutic targets (Debouck and Metcalf, 2000; Zhang et al., 2011).

Omics technologies have endowed researchers with the capacity to scrutinize samples at diverse levels, encompassing genes, transcripts, proteins, metabolites, and interaction networks, in the process of identifying new targets for drugs (Matthews et al., 2016). The integration of next-generation sequencing (NGS) technologies with computational biology is revolutionizing microbiology, enabling unprecedented insights into microbial diversity and function (Laudadio et al., 2019). The swift advancements in NGS, coupled with the concurrent bioinformatics tools, empower researchers to efficiently produce genome sequences (Jerzy, 2016; Laudadio et al., 2019) and also find utility in diverse areas such as transcriptome sequencing, metagenome sequencing, targeted sequencing or candidate gene sequencing (Wang et al., 2009; Pelizzola and Ecker, 2011; Rabbani et al., 2014; Leo et al., 2015; Jerzy, 2016).

**FIGURE 1:**
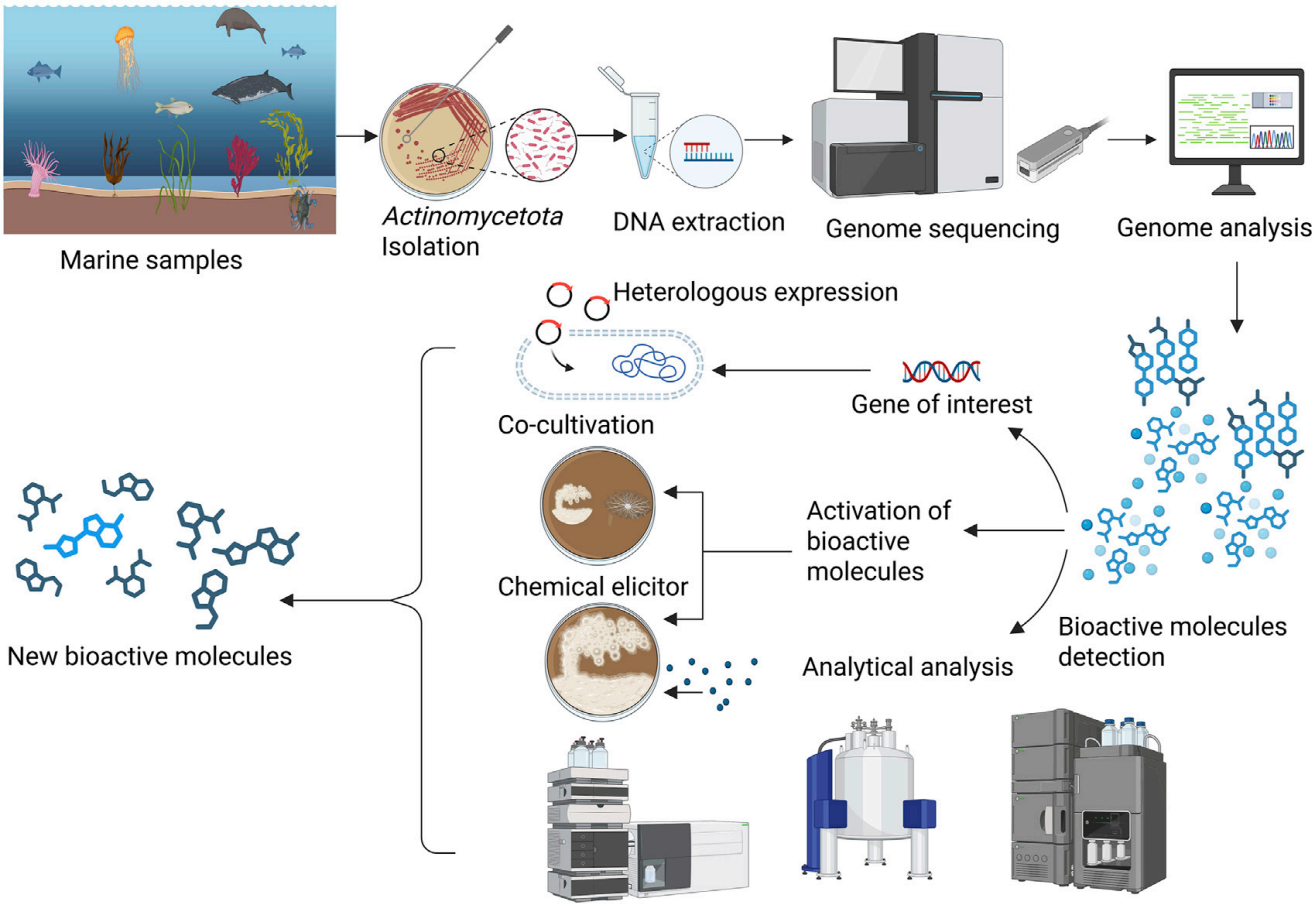
Genome and analytical approaches for the detection of new bioactive molecules from marine *Actinomycetota*.

**FIGURE 2:**
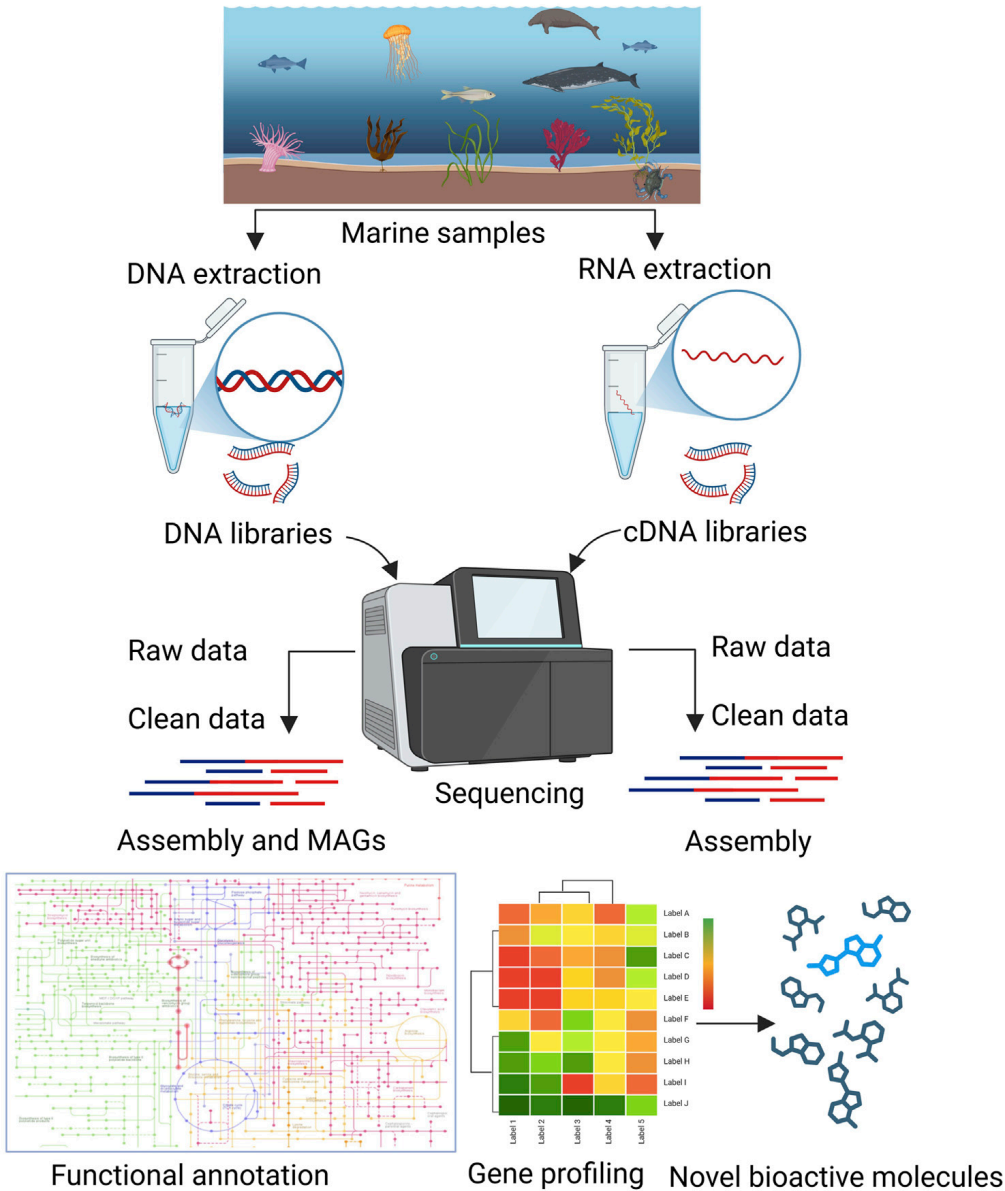
Metagenomics and transcriptomics approaches for the detection of new bioactive molecules from marine *Actinomycetota.*

In the past, various reviews have covered the discovery and development of drugs from marine *Actinomycetota* (Lam, 2006; Siro et al., 2023; Liu et al., 2024), there remains a noticeable lack of comprehensive reviews specifically addressing the application of omics-based approaches for drug discovery in this group. The present review focuses on various omics approaches used for the discovery and development of drugs from marine *Actinomycetota* (Figures 1, 2).

## Genomic approach

Since the discovery of streptomycin from *Streptomyces* (Schatz et al., 2005), this genus has received considerable attention, being a primary source of antibiotics. Traditionally, drug discovery has relied on bioactive molecule screening, followed by analytical analysis (Lee et al., 2020). In recent decades, there has been a dramatic drop in new drug development, mostly due to the repeated rediscovery of known compounds in the same ecological environments, as well as the associated cost (Belknap et al., 2020). Moreover, under laboratory conditions, microbes frequently cease secondary metabolite production, further complicating drug discovery efforts (Ohnishi et al., 2008). Additionally, there is a dearth of understanding regarding how to stimulate their biosynthesis or determine which compounds are more likely to exhibit desirable biological activities (Ohnishi et al., 2008; Augustijn et al., 2024).

Genome sequencing stands as a robust method, encompassing the complete determination of an individual’s DNA sequence and offering an intricate blueprint of their genetic composition (Satam et al., 2023). Traditional methods for finding natural compounds in microorganisms have significantly understated their capacity for biosynthesis; however, genome sequencing has uncovered a vast database of biosynthetic gene clusters (BGCs), which greatly outnumbers the number of compounds currently associated with a particular organism (Van Lanen and Shen, 2006). For instance, the model organism *Streptomyces coelicolor* is well known to produce secondary metabolites (Price et al., 1999). Genome analysis has revealed a greater number of secondary metabolites BGCs than initially anticipated (Bentley et al., 2002). Recent breakthroughs in DNA sequencing technology have resulted in a significant rise in the sequencing of *Actinomycetota* genomes (Narsing Rao et al., 2020; Li et al., 2023) and, as a result, a plethora of technologies for genome annotation and mining have emerged. The bioinformatic tool and database for the detection of BGCs such antiSMASH (Blin et al., 2019), prediction informatics for secondary metabolomes (PRISM) (Skinnider et al., 2017), NP. searcher (Li et al., 2009), DeepBGC (Hannigan et al., 2019), minimum information about a biosynthetic gene cluster (MIBiG) (Zdouc et al., 2025), natural products atlas (Poynton et al., 2025), etc., have significantly streamlined the analysis process by enabling efficient detection and characterization of bioactive compounds.

In the past few years, many marine-derived *Actinomycetota* genomes have been sequenced to evaluate their drug potential. Genome mining of marine sediment-derived *Streptomyces* sp. GMY01 revealed 28 BGCs involved in the production of flaviolin, geosmin, ectoine, class IV lanthipeptide/SflA, albaflavenone, and informatipeptin (Widada et al., 2023). Similarly, genome mining of marine sediment-derived *Streptomyces* sp. DUT11 revealed the presence of anti-complement agent (tunicamycin) and medermycin analogs, as well as new BGCs, suggesting the presence of novel lassopeptides and lantibiotics (Xu et al., 2018). Genome mining of the deep-sea-derived *Streptomyces antibioticus* OUCT16-23 revealed the presence of filipin-type polyene macrolides exhibiting antifungal activity against *Candida albicans* (Bao et al., 2022). Genome mining of marine *Streptomyces* sp. H-KF8 identified several nonribosomal peptides, leading to the design and synthesis of eight peptides, six of which showed antimicrobial activity, with two potentially disrupting membrane via a novel ion-passage mechanism (Beyer et al., 2024).

Genome analysis of *Actinomycetota* associated with marine living entities was also carried out. Genome analysis of *Streptomyces poriferorum*, a novel species isolated from a marine sponge, revealed 41 BGCs for secondary metabolites. The species showed antibacterial activity, notably against Gram-positive bacteria, including methicillin-resistant *Staphylococcus aureus* (MRSA) (Sandoval-Powers et al., 2021). Genome mining of *Streptomyces seoulensis* A01, which was isolated from a marine prawn, showed the presence of streptoseomycin (Zhang et al., 2018), while the novel species *Streptomyces poriticola*, isolated from the marine invertebrate *Porites lutea*, demonstrated notable antimicrobial properties and selective cytotoxic effects against human breast cancer MCF-7 cells, while exhibiting minimal toxicity to human dermal papilla cells (Kanchanasin et al., 2024). Furthermore, the complete genome sequence of mangrove-isolated *Streptomyces* sp. FIM 95-F1 strain revealed its ability to produce the antifungal antibiotic scopafungin (Fei et al., 2024). A combination of Illumina and PacBio sequencing was utilized to generate a high-quality, chromosome-level genome along with a plasmid for the marine *Streptomyces* sp. 891, revealing the Type II polyketide synthase (T2PKS) BGC responsible for chrysomycin production (Hu et al., 2022). Genome analysis of endophytic *Streptomyces parvulus* VCCM 22513 isolated from mangrove plant *Bruguiera gymnorrhiza* showed the presence of genes involved in mycothiol and ergothioneine biosynthesis (Quach et al., 2022). Genome analysis of *Streptomyces* sp. V17-9 isolated from seagrass showed the presence of siderophore compounds and amino acid derivatives (Kim et al., 2022). In a comparative genomic study, single-molecule real-time (SMRT) sequencing (PacBio RSII sequencing platform) of marine sponge-derived *Streptomyces* strains SM17 and SM18 enabled detailed analysis of their biosynthetic capacities. Genome mining using antiSMASH identified 20 and 26 BGCs in SM17 and SM18, respectively, many of which were either unique or showed low similarity to known clusters. Comparative analyses further revealed substantial divergence from their terrestrial relatives, not only in BGC content but also in genes linked to environmental adaptation, such as those involved in osmotic stress response and host-associated interactions. These results emphasize the impact of the marine niche on genomic diversification and point to the considerable potential for cryptic BGC activation and novel metabolite discovery (Almeida et al., 2019).

Apart from the genus *Streptomyces,* other *Actinomycetota* genera genomes were also mined to find their secondary metabolite BGCs. Genome analysis of the genus *Salinispora,* which was first described from a marine habitat (Maldonado et al., 2005), revealed many secondary metabolites like salinosporamide K from *Salinispora pacifica* (Eustáquio et al., 2011), lanthipeptide from *Salinispora* spp. (Kittrell et al., 2020), salinilactam A, and lomaiviticin from *Salinispora tropica* (Udwary et al., 2007; Kersten et al., 2013), polyketides, non-ribosomal peptides, and terpenes from *Salinispora* sp. H7-4 (Ulanova et al., 2020). Genome mining of *Janibacter limosus* P3-3-X1 from the Antarctic deep sea revealed five potential BGCs involved in secondary metabolites, including non-ribosomal peptide synthetase (NRPS), ectoine, and siderophore. The siderophore cluster may produce desferrioxamine-like iron chelators for thalassemia treatment, while a unique NRPS cluster suggests the potential for novel natural products (Su et al., 2019). Genome mining of three marine *Micromonospora* species revealed the presence of bleomycin, lymphostin, phosphonoglycan, actinomycin, alnumycin, epothilone, spinosad, syringomycin, and sioxanthin BGCs. Notably, certain BGCs exhibited species-specific distribution, highlighting the unique metabolic potential within each *Micromonospora* species (Contreras-Castro et al., 2019). *Nocardiopsis dassonvillei* RACA-4, isolated from Red Sea nudibranchs, harbors diverse BGCs for polyketides, non-ribosomal peptides, phenazine, bacteriocins, surfactins, and sactipeptides, with many showing low similarity to known clusters, indicating potential for novel natural product discovery (Elfeky et al., 2023). A list of marine-derived *Actinomycetota* gene clusters identified through genome mining are mentioned in Table 1.

**TABLE 1:** Marine-derived *Actinomycetota* gene clusters identified via genome mining.

*Actinomycetota*	Isolated from	Gene cluster related to	Sequencer/platform	References
*Nocardiopsis* sp. and *Streptomyces* spp.	Marine sediment and sponge	NRPS gene, terpenes, lassopeptide, NRPS-independent, IucA/IucC-like siderophores, PKS, lanthipeptide-class-i clusters, melanin, ectoine and others	Novaseq 6,000 Illumina	Kumar et al. (2025)
*Micromonospora* sp. SH-82	Sponge *Scopalina hapalia*	Terpene, NRPS, PKS, lanthipeptide, lipolanthine, NRP metallophore, phenazine, siderophore, and resorcinol	Oxford Nanopore GridIon and Illumina HiSeq 2,500	Ramesh et al. (2024)
*Streptomyces malaysiensis* HNM0561	Marine sponge	Malaymycin and mccrearamycin E	PacBio RS II and Illumina HiSeq 4,000	Zhu et al. (2022)
*Janibacter indicus* YB324	Marine sediment	Non-ribosomal peptide synthetase (NRPS) ectoine, siderophore and terpenes	PacBio Sequel	Pei et al. (2021)
*Streptomyces* sp. SCSIO 03032	Deep-sea sediment	Piericidins, heronamides and spiroindimicins/indimicins/lynamicins	PacBio RS II	Ma et al. (2021)
*Micromonospora craniellae*	*Craniella* species sponge	Nonribosomal peptides, polyketides, terpene, siderophore, etc	HiSeq and PacBio RSII/Sequel system	Yang et al. (2021)
*Salinispora* sp. H7-4	Deep-sea sediment	Polyketides, nonribosomal peptides, and terpenes	HiSeq 4,000	Ulanova et al. (2020)
*Streptomyces* sp. DUT11	Marine sediment	Tunicamycins, ectoine, siderophore, bacteriocin, butyrolactone, novel lassopeptides and lantibiotics	Pacbio RS	Xu et al. (2018)
*Micromonospora echinospora* SCSIO 04089	Marine sediment	Nenestatin A	Not mentioned	Jiang et al. (2017)
*Brachybacterium* sp. P6-10-X1	Deep-sea sediments	Siderophore, ectoine, terpene, and PKS gene	Illumina Hiseq 4,000 and PacBio RSII	Zhao et al. (2017)
*Micromonospora* sp. DSW705	Deep seawater	PKS, NRPS and hybrid PKS/NRPS gene clusters	2,1,	Komaki et al. (2016)
*Micromonospora* sp. RL09-050-HVF-A	Marine sediment	Lobosamides A–C	Single molecule realtime sequencing technology (Pacific Biosciences)	Schulze et al. (2015)
*Sciscionella* SE31	Intertidal sediment	Nonribosomal peptides, polyketides, and oligosaccharides	Illumina HiSeq 2000	Teo et al. (2015)
*Streptomyces* sp. AA0539	Marine sediment	siderophores, terpenes, lantibiotic, PKS, NRPS, nucleosides, ectoine, and hybrid NRPS/PKS	Roche 454 genome sequencer FLX	Xiong and Wang, (2012)

## Genome-guided combinatorial approach

Genome sequencing has revealed that many microbial BGCs remain inactive under standard culture conditions, limiting access to novel secondary metabolites (Onaka, 2017). Heterologous expression, a method involving the transfer of gene clusters into a different, more amenable host, has proven effective for activating these silent pathways and enhancing novel metabolite production. This approach played a crucial role in uncovering the hidden metabolic potential of microbes for natural product discovery (Yang et al., 2020). The integration of genome mining with heterologous expression has successfully activated silent BGCs in numerous marine-derived *Actinomycetota*, enabling the discovery of novel secondary metabolites. Genome mining of the marine *S. seoulensis* A01 enabled the identification of a giant Type I PKS gene cluster (*asm*). When this BGC was constructed and expressed in “*Streptomyces lividans*” SBT18, ansaseomycin A and B were produced, which were active against the leukemia cell line (Liu et al., 2019). Genome mining of the sponge-associated *Streptomyces* sp. DSS69 uncovered putative genes involved in macrolactam biosynthesis. Subsequent cloning and heterologous expression of these genes in “*S. lividans”* GX28 led to the discovery of weddellamycin, an antibacterial compound exhibiting potent activity against a range of Gram-positive bacteria, including MRSA, as well as antifungal activity against *C. albicans* and cytotoxic effects on various cancer cell lines (Chen et al., 2024). Recently, the integration of genome mining and heterologous expression led to the discovery of two novel tricyclic diterpenes, ostamycins A and B, from the deep-sea-derived *Streptomyces amphotericinicus* DS22–01, both exhibiting inhibitory activity against the Influenza A virus (Hou et al., 2025).

Along with genomic and heterologous expression approaches, cultivation conditions and analytical methods were also used to activate BGCs. Genomic analysis of marine *Actinoalloteichus* sp. AHMU CJ021 revealed 22 BGCs, including a dormant caerulomycin A (CRM A) pathway. Activation of CRM A was achieved via gentamycin-guided ribosome engineering, with further enhancement through UV mutagenesis and intracellular riboflavin optimization. Medium optimization using response surface methodology showed that controlled carbon feeding and high organic nitrogen levels, with limited inorganic nitrogen, significantly improved CRM A yield (Xie et al., 2020). Integrating NMR-based metabolomics with genomic analysis has proven effective for natural product discovery in marine-derived actinobacteria. In *Streptomyces* sp. S063, this approach revealed a novel NRPS gene cluster and identified cyclic decapeptides with moderate anticancer activity (Huang et al., 2023). A study used a combination of genome and MS/MS analysis to investigate the biosynthetic potential of a rare actinobacterium (*Micromonospora aurantiaca* sp.01) isolated from a mangrove habitat. Analysis of its genome revealed 21 secondary metabolite BGCs responsible for antibiotic production. Using guided MS/MS analysis, one of the predicted antibiotics, kanamycin, was identified (Hu et al., 2020). *Streptomyces* sp. MP131-18, isolated from marine sediment, was subjected to integrated genomic and metabolomic profiling. Genome mining via antiSMASH uncovered 36 BGCs associated with the production of 18 diverse classes of secondary metabolites, indicating a rich and varied metabolic capacity. Complementary metabolomic analyses led to the identification of bisindole pyrrole compounds, including lynamicins and spiroindimicins, which showed antibacterial activity against *Bacillus subtilis (* Paulus et al., 2017 *)*.

Eliciting bacterial cells using external signals, whether biological (such as co-cultivation with other microbes) or chemical (like small molecule inducers), is a strategic approach to activate silent or poorly expressed BGCs responsible for antibiotic production (Abdelmohsen et al., 2015). Co-culturing different microbial species is a simple yet powerful approach to activate silent BGCs (Kim et al., 2021). When coupled with genome analysis, which identified cryptic BGCs, co-culture served as a targeted strategy to activate biosynthetic potential. This method not only mimics natural ecological stressors like interspecies competition and nutrient limitation but also enables real-time assessment of induced metabolite (Kim et al., 2021). A study highlights how co-cultivating a marine-derived *Streptomyces* sp. PTY087I2 with human pathogens (*B. subtilis*, methicillin-sensitive *S. aureus*, MRSA, and *Pseudomonas aeruginosa*) effectively activates silent BGCs, leading to the production of novel antibiotic compounds. Genome analysis of *Streptomyces* sp. PTY087I2 revealed 37 BGCs with high biosynthetic potential; however, monoculture conditions failed to induce significant metabolite expression. In contrast, co-culture conditions led to the enhanced production of granaticin, granatomycin D, dihydrogranaticin B, and related analogues, significantly boosting antimicrobial activity against Gram-positive pathogens (Sung et al., 2017). Similarly, genome and co-culture analysis of marine invertebrate-associated bacteria, specifically *Micromonospora* and *Rhodococcus* species, led to the discovery of the novel antibiotic keyicin. The genome of *Micromonospora* was found to contain the genes responsible for keyicin biosynthesis, whereas *Rhodococcus* did not. Co-culture experiments showed that *Micromonospora* sp. exposure to *Rhodococcus* sp. derived signals triggered *Micromonospora* to enhance genes involved in the keyicin biosynthetic pathway. The resulting compound exhibited antibacterial activity, particularly against selective Gram-positive bacteria, including *Rhodococcus* and *Mycobacterium* species (Adnani et al., 2017).

Chemical elicitation, on the other hand, uses synthetic compounds like inorganic substances, heavy metals, and rare earth elements to trigger metabolic changes by activating specific defence pathways with varying intensity (Abdelmohsen et al., 2015). The combination of genome analysis with biological and chemical elicitation proved effective in revealing hidden biosynthetic capabilities. A recent study investigated elicitation strategies to enhance antibacterial metabolite production in Antarctic actinobacterial strains from soil, marine water, and sediments. By employing MS/MS-based metabolomics and genome mining, strains were cultivated under different nutrient conditions and elicitors such as lipopolysaccharide, sodium nitroprusside, and co-culture. While all treatments activated biosynthetic pathways, strain-specific responses varied depending on culture medium composition (Núñez-Montero et al., 2020).

## Metagenomics and transcriptomics

The exploration of microbial diversity and function in natural environments has been greatly enhanced by high-throughput sequencing technologies. Metagenomics, which involves sequencing DNA extracted directly from environmental sources, enables researchers to study entire microbial communities without the need for culturing individual species. This approach provides a broad view of the taxonomic composition and metabolic capabilities present within complex microbiomes (Akaçin et al., 2022). Complementing this, transcriptomics focuses on the analysis of RNA transcripts, offering insights into gene expression patterns under specific environmental or physiological conditions. By capturing active transcriptional responses, transcriptomic studies reveal which genes were being expressed and regulated, providing a functional perspective on microbial activity (Aplakidou et al., 2024). Through integrated metagenomic and transcriptomic analyses, researchers have been able to uncover a diverse array of BGCs and regulatory pathways involved in the synthesis of potentially therapeutic molecules from marine *Actinomycetota*. Metagenomic studies targeting the deep chlorophyll maximum of the Mediterranean Sea have led to the recovery of four genomes belonging to marine *Actinobacteria*, specifically within the *Acidimicrobiales* order. These represent the first genomic insights into marine representatives of this group. Among the four genomes, one was found to carry a gene coding for a rhodopsin-like protein, exhibiting closest similarity to a freshwater *Acidimicrobiales* species. The associated rhodopsin gene cluster displayed unique features distinct from previously known variants, prompting the designation of a new subgroup referred to as acidirhodopsins (Mizuno et al., 2015). An integrative omics study combining transcriptomics and proteomics with parallel reaction monitoring has elucidated the antifungal mechanism of antifungalmycin B, a bioactive compound from the marine *Streptomyces hiroshimensis*. These findings reveal that antifungalmycin B inhibits *Talaromyces marneffei* by disrupting organic acid biosynthesis and impairing critical cellular energy metabolism pathways. Such dual interference undermines metabolic homeostasis in the pathogen, enhancing antifungalmycin B antifungal activity (Li et al., 2025). The marine-derived *Streptomyces olivaceus* SCSIO T05 has emerged as a promising source of antifungal compounds, particularly in the context of targeting virulence traits in *C. albicans* (inhibiting the formation of hyphae and biofilms). Transcriptomic analysis, supported by real-time PCR, revealed that these effects were mediated through the downregulation of genes associated with filamentation and cell adhesion. This gene expression modulation disrupts essential morphogenetic and adhesion pathways, suggesting that the compound impairs fungal pathogenicity by targeting regulatory networks rather than directly killing the fungal cell. These findings highlight the potential of transcriptomics-guided discovery in identifying novel anti-virulence strategies against fungal pathogens (Meng et al., 2019). A study employed comparative transcriptomics to analyze BGC activity across four closely related *Salinispora* strains. The results showed that about half of the BGCs were actively expressed at levels likely sufficient for metabolite detection. By comparing similar clusters across strains, specific regulatory genes potentially responsible for BGC silencing were identified. These previously undetected regulatory variations emphasize the significance of transcriptomic approaches in uncovering hidden metabolic potential. The presence of conserved but transcriptionally inactive BGCs across multiple strains suggests they may be subject to distinct regulatory mechanisms or that gene silencing serves an evolutionary function. Combining transcriptomic data with metabolomics allowed to associate the production of salinipostins (Amos et al., 2017).

## Conclusion and future perspectives

Marine *Actinomycetota* possess exceptional biosynthetic potential, producing a wide range of bioactive secondary metabolites with significant pharmaceutical relevance. Their unique metabolic capabilities underscore their value as a promising source for novel drug discovery and therapeutic development. However, the exploration of this microbial group has so far barely scratched the surface. For years, researchers depended on conventional isolation and bioactivity-guided screening methods, which, although fruitful to a degree, often led to the rediscovery of previously known compounds. This recurring outcome underscores a critical limitation: only a small portion of marine microbial life has been cultured and studied, leaving the vast majority untapped, much like seeing only the tip of an iceberg while the bulk remains submerged and mysterious. With the advent of omics technologies, this landscape is beginning to change. Genome sequencing has opened the door to BGCs from microbes, offering clues to potentially novel compounds. Metagenomics has proven even more transformative, granting access to the genetic blueprints of uncultivable microbes directly from environmental samples, an essential step toward revealing the hidden biosynthetic capacity of marine ecosystems. Meanwhile, transcriptomic analyses help unravel the gene expression patterns that regulate secondary metabolism, and metabolomics allows researchers to profile complex chemical mixtures and associate them with specific metabolic pathways or gene clusters. Complementing these molecular tools are powerful analytical techniques that bring chemical insights into sharper focus. High-resolution mass spectrometry and nuclear magnetic resonance spectroscopy are critical for structure elucidation and dereplication, helping distinguish novel compounds from known ones. Liquid chromatography-mass spectrometry (LC-MS) enables detailed metabolic profiling, allowing researchers to connect metabolomic data with genomic predictions. These techniques, when used in tandem with bioinformatics tools and databases, enhance the precision and speed of natural product discovery. Despite these advancements, many challenges remain. A substantial number of BGCs identified in genome data are still uncharacterized or remain silent under laboratory conditions. Future efforts should focus on improving the functional annotation of these gene clusters through advanced computational and experimental methods, and on developing more efficient ways to activate and study these cryptic biosynthetic pathways. Equally important is the exploration of lesser-known marine habitats such as deep-sea trenches, hydrothermal vents, polar seas, and marine symbiont communities that likely harbor microbial species with entirely novel metabolic capacities. The full potential of marine *Actinomycetota* will only be realized through integrated, multidisciplinary efforts that combine omics-driven discovery, chemical analytics, systems biology, and ecological exploration. As researchers continue to piece together this complex puzzle, each breakthrough will bring us closer to uncovering new classes of bioactive molecules with the potential to address critical challenges in medicine, particularly the growing crisis of antibiotic resistance and the urgent need for innovative therapeutics.
